# Incidence of *Cronobacter* spp. Infections, United States, 2003–2009

**DOI:** 10.3201/eid2009.140545

**Published:** 2014-09

**Authors:** Mary E. Patrick, Barbara E. Mahon, Sharon A. Greene, Joshua Rounds, Alicia Cronquist, Katie Wymore, Effie Boothe, Sarah Lathrop, Amanda Palmer, Anna Bowen

**Affiliations:** Centers for Disease Control and Prevention, Atlanta, Georgia, USA (M.E. Patrick, B.E. Mahon, S.A. Green, A. Bowen);; Minnesota Department of Health, St. Paul, Minnesota, USA (J. Rounds);; Colorado Department of Public Health and Environment, Denver, Colorado, USA (A. Cronquist);; California Emerging Infections Program, Oakland, California, USA (K. Wymore);; Tennessee Department of Health, Nashville, Tennessee, USA (E. Boothe);; New Mexico Emerging Infections Program, Albuquerque, New Mexico, USA (S. Lathrop);; Maryland Department of Health, Baltimore, Maryland, USA (A. Palmer)

**Keywords:** *Cronobacter sakazakii*, *Enterobacter sakazakii*, infants, FoodNet, epidemiology, United States, bacteria

## Abstract

During 2003–2009, we identified 544 cases of *Cronobacter* spp. infection from 6 US states. The highest percentage of invasive infections occurred among children <5 years of age; urine isolates predominated among adults. Rates of invasive infections among infants approximate earlier estimates. Overall incidence of 0.66 cases/100,000 population was higher than anticipated.

*Cronobacter* spp. are gram-negative bacteria mainly perceived to cause serious infections in infants (*1*). Cases are most common among newborns or young infants; estimated mortality rates are as high as 80% (*2*). Infections also occur in older children and adults (*3*). In adults, *Cronobacter* spp. cause septicemia, pneumonia, osteomyelitis, wound infections, and splenic abscesses (*4*).

Little is known about reservoirs or routes of transmission other than ingestion of contaminated powdered infant formula (*5**–**7*). However, organisms have been isolated from other foods and environmental sources, and infections have occurred among persons who did not consume or handle formula (*8*,*9*).

In the United States, the incidence of *Cronobacter* spp. infection is unknown, but evidence suggests it is low. In 1998, only 1 case was found among 10,660 hospitalized infants of low birth weight (*10*). In 2002, the Foodborne Diseases Active Surveillance Network (FoodNet) estimated an incidence of 1 case per 100,000 infants (*11*). However, these estimates might be unreliable because populations studied were small and the disease is rare. There is no national surveillance for *Cronobacter* spp. infections; such infections are reportable only for infants in Minnesota. To increase understanding of the public health effects and demographic distribution of *Cronobacter* spp. infections, we investigated incidence of laboratory-confirmed infection and characteristics of infected persons.

## The Study

FoodNet is a collaborative program among the Centers for Disease Control and Prevention, 10 state health departments, the US Department of Agriculture, and the Food and Drug Administration. FoodNet conducts active, population-based surveillance for selected laboratory-confirmed enteric infections. In 2010, clinical laboratories within FoodNet were asked to query records for all laboratory-confirmed isolations of *Cronobacter* spp. (formerly *Enterobacter sakazakii*) reported from January 1, 2003, through December 31, 2009, or for as many months in this period as was feasible. These isolations are subsequently referred to as *Cronobacter* cases. Participating laboratories were in California, Colorado, Maryland, Minnesota, New Mexico, and Tennessee. We recorded information on patient age, isolation site, and date (month and year) of specimen collection. We defined invasive infection as isolation of the organism from blood or cerebrospinal fluid. We conducted descriptive analyses and calculated incidence rates within age groups and overall by using Excel (Microsoft Corp., Redmond, WA, USA) and SAS version 9.3 (SAS Institute, Cary, NC, USA).

For incidence calculations, we estimated the number of persons served by each laboratory by calculating the proportion of isolates of other enteric pathogens (*Campylobacter*, *Listeria*, *Salmonella*, *Vibrio*, and *Yersinia* spp. and Shiga toxin–producing *Escherichia coli*) that each laboratory reported to FoodNet, by age group, for each year that the laboratory was surveyed. We applied this proportion to US census data for that age group, year, and FoodNet site and summed values from all laboratories to obtain an age-group denominator for 2003–2009. We then calculated age-specific incidence rates by dividing the number of *Cronobacter* cases for each age group by the overall age-group denominator and multiplying by 100,000. Similarly, we calculated the overall incidence rate by dividing the total number of *Cronobacter* cases by the sum of the age-group denominators and multiplying by 100,000.

**Figure 1:**
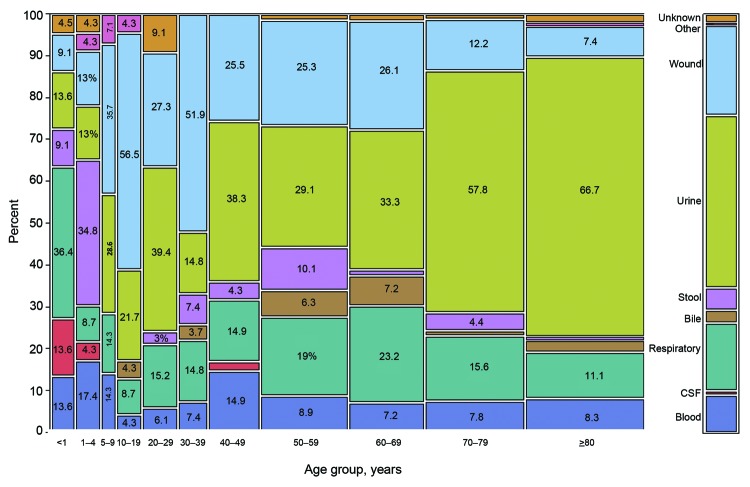
Isolations of *Cronobacter* spp., by specimen source and patient age group, Foodborne Diseases Active Surveillance Network (FoodNet), 2003–2009. Data are based on a sample from laboratories in 6 states (California, Colorado, Maryland, Minnesota, New Mexico, and Tennessee) in the FoodNet catchment area and are reported for 535 of 544 patients (age information missing for 9 patients). Width of the column is proportional to the number of isolations. CSF, cerebrospinal fluid.

We identified 544 *Cronobacter* cases from participating laboratories at 6 FoodNet sites. The overall median patient age was 59 years (range 1 day–100 years); by state, median ages ranged from 52 years (Tennessee) to 71 years (Colorado). Of the 544 patients, 198 (37%) were >70 years of age and 22 (4%) were infants <1 year of age. *Cronobacter* spp. was most frequently isolated from urine (221 [41%] isolates); wound, abscess, or surgical site (115 [21%]); respiratory tract (sputum, pharyngeal swab, or tracheal aspirates) (87 [16%]); blood (51 [ 9%]); feces (31 [6%]); bile (16 [3%]); cerebrospinal fluid (5 [1%]), and other or unknown sources (18 [3%]). The highest percentage of invasive infections occurred among infants (6 [27%] of 22) and children 1–4 years of age (5 [22%] of 23), and isolates from urine predominated among those in most adult age categories (Figure 1). Other than a slight increase in the number of isolations in April, no seasonal patterns were detected.

**Figure 2:**
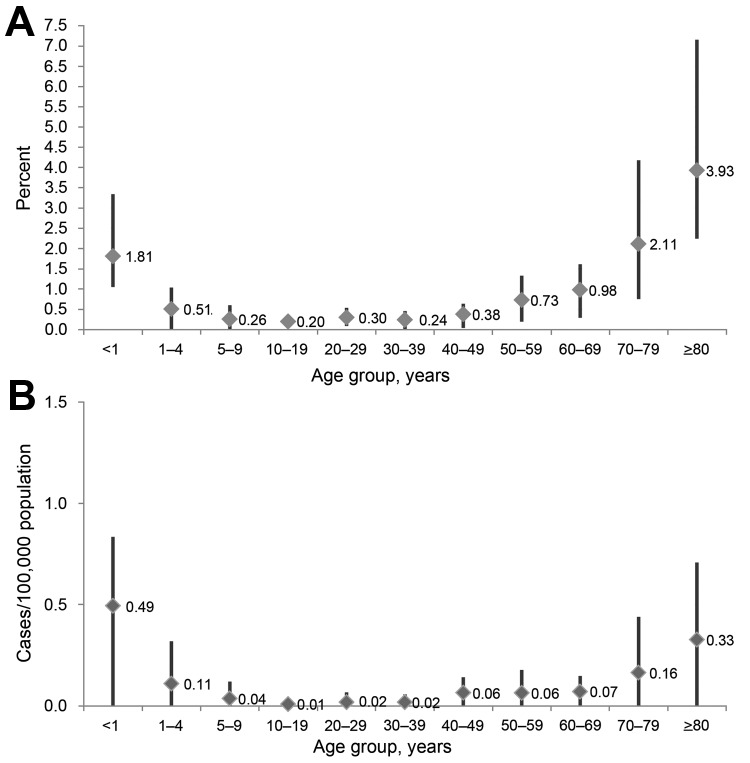
A) *Cronobacter* spp*.* incidence rates, by age group (overall and range by site) in the Foodborne Diseases Active Surveillance Network (FoodNet), 2003–2009. B) *Cronobacter spp.* incidence rates for invasive isolates by age group (overall and range by site), FoodNet, 2003–2009. Data are based on a sample from laboratories in 6 states (California, Colorado, Maryland, Minnesota, New Mexico, and Tennessee) in the FoodNet catchment area and are reported for 535 of 544 patients (age information missing for 9 patients).

The overall incidence rate was 0.66 cases per 100,000 population; rates varied by age, state, and invasiveness. The highest rates occurred among persons >80 years of age (3.93 cases/100,000 population), followed by persons 70–79 years of age (2.11) and infants (1.81) (Figure 2). Rates of invasive infection were 0.07 cases per 100,000 population overall and were highest among infants (0.49) and persons >80 years of age (0.33) (Figure 2, panel A). Overall incidence rates among infants were highest in Minnesota, and rates among persons >80 years of age were highest in Colorado. Rates of urinary tract isolation were 0.27 isolates per 100,000 population overall and highest among persons >80 (2.62) and 70–79 (1.22) years of age.

This analysis provides population-based incidence rates of *Cronobacter* spp. infection for all age groups in the United States. Overall rates were similar to those reported for vibriosis and yersiniosis (*12*). The rates for infants in our study were similar to those previously estimated for infants in the United States (*11*). The rates of bacteremia that we found among infants and older adults in the United States were higher than those reported in the Philippines and the Netherlands and similar to those reported in the United Kingdom (*13*,*14*).The higher rates for Minnesota might be explained by the reporting requirements in that state; however, high rates among adults in other states suggest that the geographic variation may be real.

Although rates were highest among persons at age extremes, we documented isolations for persons in all age groups and from a variety of clinical specimen sources. Although infants accounted for only a small percentage of isolates, they also accounted for the highest rate of invasive infections. More than a third of isolates from infants came from the respiratory tract and might represent colonization rather than infection. Rates for adults >80 years of age were higher than rates for any other age group and twice as high as rates for infants; however, we lack information to distinguish infection from colonization.

Half of the isolates in our survey came from urine, and these were mainly from older adults. Although others have documented urinary tract infections caused by *Cronobacter* spp. *(**3**,**15**),* our data suggest that urine might be a more common site of infection than previously thought. Studies are needed to understand the contribution of *Cronobacter* spp. to the prevalence and costs associated with urinary tract infections, which are common in older adults.

Our findings are subject to limitations. Only a third of laboratories that participate in FoodNet provided data for this study, and reporting varied by state and year. Our sample might not be representative of the states surveyed or the United States as a whole. Because we did not review charts or interview patients, we did not have information on underlying conditions, clinical signs and symptoms, patient sex, or possible exposures. Isolates were not available for speciation or subtyping.

## Conclusions

Although rates of invasive *Cronobacter* spp. infections among infants in our study approximated earlier estimates, overall rates of isolation of *Cronobacter* spp. from ill persons in the United States were higher than we anticipated, and the very young and very old were disproportionately affected. A substantial proportion of isolates came from the urinary tract, raising questions about risk factors for transmission and clinical manifestations. Routine, systematic surveillance and special studies will be essential for understanding these findings, identifying reservoirs of infection and vehicles of transmission, and developing effective prevention and control measures.
